# Is it possible to extract primary maxillary molars without palatal injection?: a controlled clinical trial

**DOI:** 10.1007/s00784-024-05565-x

**Published:** 2024-02-26

**Authors:** Esra Ceren TUĞUTLU, Kevser SANCAK

**Affiliations:** 1 Department of Pedodontics, Faculty of Dentistry, Ankara Yıldırım Beyazıt University, Ankara, Türkiye; 2 Department of Oral and Maxillofacial Surgery, Faculty of Dentistry, Ankara Yıldırım Beyazıt University, Ankara, Türkiye

**Keywords:** Local anesthesia, Pain perception, Tooth extraction, Injections, Articaine

## Abstract

**Objectives:**

The absolute necessity of a palatal injection for the extraction of primary maxillary molars has never been explored, despite the fact that it is widely known that children do not tolerate local anesthetic injections into the palatal tissue well. The aim of this study was to compare separately the perception of pain in the absence of palatal injection after anesthesia and maxillary primary molar tooth extraction using different anesthetic solutions and different post-anesthetic waiting times.

**Materials and methods:**

A single-blinded randomized controlled study was conducted in 78 participants (26 patients with palatal anesthesia (the control groups), and 26 patients with 5 min and 26 patients with 8 min post-anesthetic waiting time without palatal anesthesia (the study groups)). Subjective experiences of pain were evaluated separately after anesthesia and tooth extraction using the Visual Analog Scale (VAS) and the Wong-Baker Faces Pain Rating Scale (WBS).

**Results:**

In terms of VAS scores obtained following administration of anesthesia, there was a statistically significant difference between the groups (*p*<0.05). VAS pain scores were reported to be lower in the groups without palatal anesthesia than in the groups with palatal anesthesia. No statistically significant difference was observed in VAS and Wong-Baker scores after tooth extraction between the groups with and without palatal anesthesia (*P*>0.05).

**Conclusions:**

While the pain reported following administration of anesthesia was found to be higher in the groups receiving palatal anesthesia, no difference was found between the groups in the pain reported after tooth extraction.

**Clinical relevance:**

Extraction of maxillary primary molars is possible without palatal injection by injecting 4% articaine or 2% lidocaine into the buccal vestibule of the tooth with a waiting time of 5 or 8 min.

## Introduction

The American Academy of Pediatric Dentistry (AAPD) recognizes that the pain experienced by children and adolescents due to inadequate pain management during dental procedures can have significant physical and psychological consequences for the patient [1]. Tooth extraction is an invasive procedure in which pain control is critical for reducing anxiety and improving children’s cooperation [2]. The key to avoiding pain during this procedure is the use of local anesthesia. Yet the local anesthetic injection itself can also trigger anxiety, which causes pain and leads to difficulties in behavior management [3]. Palatal injections in particular are known to be poorly tolerated by the majority of both adults and pediatric patients [4]. For this reason, many studies have investigated whether maxillary teeth extraction can be performed without palatal anesthesia in the adult patient group, and it has been concluded that buccal infiltration anesthesia is sufficient for permanent maxillary permanent teeth extraction [5–8]. However, this issue has never to our knowledge been investigated in the pediatric patient group.

Furthermore, in several studies, various different waiting times have been investigated when performing maxillary molar tooth extraction without palatal anesthesia. In many studies, it was reported that a 5-minute waiting time was sufficient for the anesthetic agent to spread [5, 6, 9]. Sharma et al. [7] reported that a waiting period of 6–7 min eliminated the need for palatal injection of articaine and lidocaine infiltration, while Sekhar et al. [10] found that this required a period of 8 min. Lima-Júnior et al. [11] suggested that with a 10-minute waiting time, a painless extraction could be performed with only buccal anesthesia.

In the last century, local anesthetics formed the basis of pain control in dentistry and various solutions were used. Lidocaine, which was first used in 1948, is considered the gold standard of local anesthetics and is the most widely used injectable amide local anesthetic agent [12]. Articaine hydrochloride is an anesthetic solution first produced by H. Rusching et al. in 1969; it acts similarly to other amide anesthetics but has several advantages over them due to differences in chemical conformation, including higher intraneural concentrations, greater longitudinal diffusion, and better conduction blockading [13].

Assessing children’s pain is important both for planning and determining the effectiveness of dental treatments. The assessment of pain, whether acute or chronic, should be simple, practical and as objective as possible. Because pain is a subjective experience, self-report of the intensity of pain is the preferred method of assessment [14]. A wide variety of pain rating scales are used to assess acute pain, including the Visual Analog Scale (VAS), the Descriptive Pain Scale, the Faces Pain Scale, the Numerical Pain Scale and the Analog Chromatic Scale. One of these scales, the Wong-Baker Faces Pain Rating Scale (WBS), has undergone extensive psychometric evaluation and is currently considered suitable for use in children aged three to eighteen years [15]. Another frequently used scale is the VAS, which is also known to give reliable results in the assessment of acute pain in children over eight years of age [16].

The hypothesis of this study was that painless extraction can be performed using only buccal infiltration anesthesia via both articaine and lidocaine. The patient’s perception of pain may not change or decrease in primary maxillary molar extractions without palatal anesthesia. The aim of this study was thus (1) to evaluate the level of anesthesia administered and the post-extraction perception of pain, and (2) to determine which local anesthetic solution and post-anesthetic waiting time were more successful in maxillary primary teeth extraction in children who did not undergo palatal anesthesia.

## Materials and methods

### Ethics and consent to participate

Ethics committee approval was obtained from the Ethics Committee of the Faculty of Medicine, at Ankara Yıldırım Beyazıt University (Date: 27.04.2022, No: 06). The study was registered retrospectively at ClinicalTrials.gov (ClinicalTrials.gov identifier, NCT06025825). The Declaration of Helsinki and good clinical practices were followed during the study. Before starting the study, the patients and their families were informed about its content, and written informed consent was obtained from the parents. This prospective, randomized controlled, single-blind trial was conducted in accordance with the CONSORT (Consolidated Standards of Reporting Trials) statement [17].

### Patient selection

**Fig. 1:**
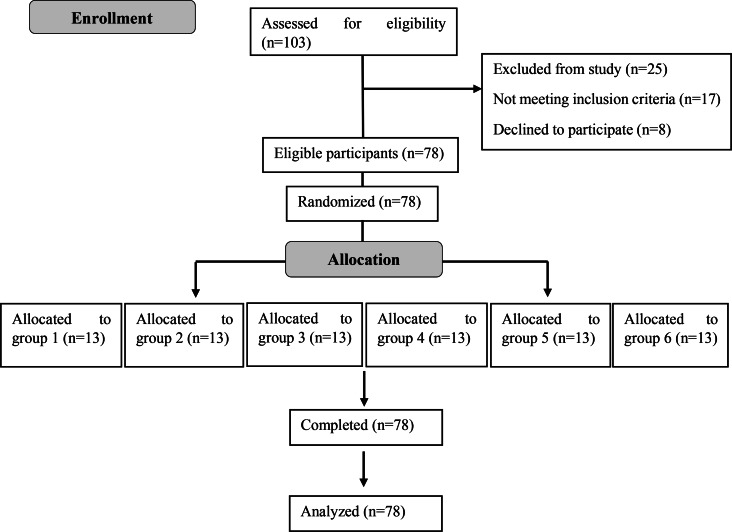
Flow diagram

To estimate the sample size, power analysis was performed with the G*Power 3.1 program, with reference to study of Bataneih et al., and with a 0.52 effect size, 95% power and 5% error level [5]. Patients aged 8–12 years, of both genders, who attended the Faculty of Dentistry Hospital at Ankara Yıldırım Beyazıt University for a routine dental examination and treatment, and who had at least one maxillary molar tooth extraction indication, were included in the study. Children with any chronic/systemic disease, acute toothache, history of tooth extraction, allergy to benzocaine, lidocaine or articaine, and Frankl’s 1 or Frankl’s 2 behavior ratings were excluded from the study. The CONSORT flow diagram (Fig. 1) depicts the enrollment, randomization, allocation, and completion of patients in groups. Patients who had at least one-third of the tooth root for which extraction was indicated were included in the study.

The groups were determined as follows:

Group 1. Buccal infiltration anesthesia + palatal infiltration anesthesia (with articaine HCl) (Control group).

Group 2. Buccal infiltration anesthesia (5 minutes’ waiting time after anesthesia with articaine HCl).

Group 3. Buccal infiltration anesthesia (8 minutes’ waiting time after anesthesia with articaine HCl).

Group 4. Buccal infiltration anesthesia + palatal infiltration anesthesia (with lidocaine HCl) (Control group).

Group 5. Buccal infiltration anesthesia (5 minutes’ waiting time after anesthesia with lidocaine HCl).

Group 6. Buccal infiltration anesthesia (8 minutes’ waiting time after anesthesia with lidocaine HCl).

### Study procedures

All anesthesia administration and tooth extractions were performed by the same pediatric dentist, who had 10 years of clinical experience. Before the solution was administered, the injection site was dried with a cotton and Ultracare 20% benzocaine gel (Ultradent Products Inc., South Jordan, UT, USA) was applied to the prepared injection site for 1 min. Following this, lidocaine hydrochloride 2% containing 0.0125 mg/ml epinephrine with Jetocain (Adeka İlaç Sanayi ve Ticaret A.Ş, İstanbul, Türkiye) or articaine hydrochloride 4% containing 0.012 mg epinephrine hydrochloride with Ultracaine D-S (Sanofi Aventis Deutschland GmbH, Frankfurt, Germany) (0.8 cc for buccal and 0.2 cc for palatal infiltration anesthesia) was injected through a Morita 30G dental needle using a Paroject intraligamental syringe in the mucobuccal fold area closest to the extracted tooth. After the local anesthesia applications, tooth extractions were performed after waiting 5 min for groups 1, 2, 4 and 5, and after 8 min for groups 3 and 6.

**Fig. 2:**
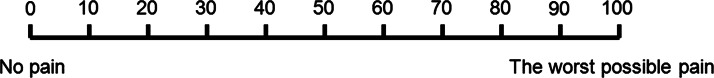
Visual Analog Scale (VAS)

**Fig. 3:**
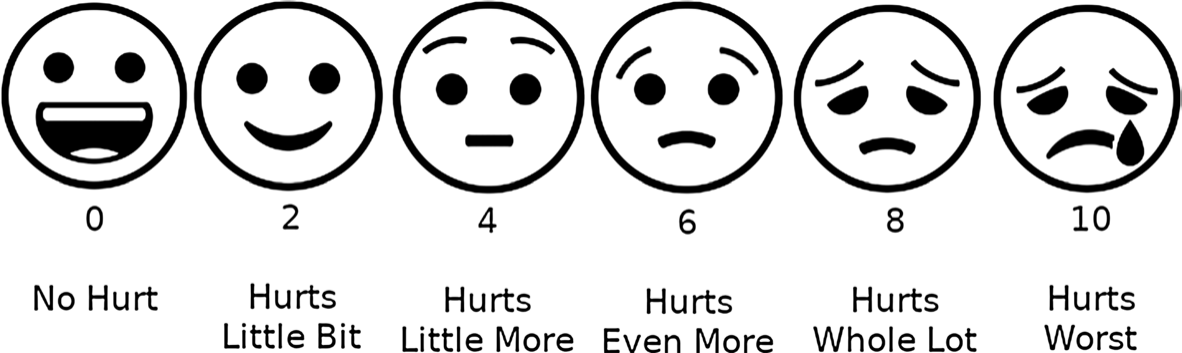
Wong-Baker Faces Pain Rating Scale (WBS)

The VAS and Wong-Baker scores of the participants were recorded separately following the administration of local anesthesia and tooth extractions. In order to determine the scores, the participants were asked the following questions:

1. “‘0’ represents no pain and ‘100’ represents the worst possible pain. Can you give a score for the pain you feel?” (for the VAS) (Fig. 2).
2. “These faces are ranked from ‘happy’ to ‘unhappy’ and show how much it hurts. Can you point to the face that shows how much pain you are feeling?” (for the WBS) (Fig. 3).

All questions were asked by a single, non-operator evaluator (KS) who was blinded to the study groups.

### Statistical analysis

The analysis of the data was performed with the IBM SPSS 26.0 program (IBM Corporations, Armonk, New York, USA). Descriptive statistics were used for discrete numerical variables and were expressed as mean ± standard deviation or median (minimum-maximum), while categorical variables were expressed as number of cases and percentage (%). Whether the distribution of discrete numerical variables was close to normal was investigated using the Kolmogorov-Smirnov test. The significance of the difference between the groups in terms of mean values was evaluated with the Kruskal-Wallis H test. If the Kruskal-Wallis H test results were found to be significant, the group(s) causing the difference was/were determined using the Mann-Whitney U test. In addition, comparisons between groups using articaine and lidocaine were made with the Mann-Whitney U test. The statistical significance level was accepted as *P* = 0.05.

## Results

The overall study sample consisted of 78 patients, 40 (51.3%) females and 38 (48.7%) males, and their ages ranged from 8 to 12 years with a mean of 9.34 (± 1.20) years. The differences in gender and age distribution between the six groups were statistically insignificant (*p* = 0.71 and *p* = 0.82). Also, there were no statistically significant differences between the six groups in terms of Frankl scores (*p* = 0.93) and the distribution of extracted teeth (*p* = 0.09).

The distribution of Wong-Baker scores after local anesthesia according to groups are given in Table 1. In terms of post-anesthesia Wong-Baker scores, there was no statistically significant difference between the groups (*p* = 0.10).

**Table 1:** Distribution of Wong-Baker scores according to groups after local anesthesia

Wong-Baker Scores	Mean ± SD	P Value
Group 1 (control group with articaine)	1.92 ± 1.44	0.10
Group 2 (buccal infiltration anesthesia with articaine/5-min. waiting time)	2.15 ± 1.06	
Group 3 (buccal infiltration anesthesia with articaine/8-min. waiting time)	1.76 ± 1.30	
Group 4 (control group with lidocaine)	2.53 ± 1.39	
Group 5 (buccal infiltration anesthesia with lidocaine/5-min. waiting time)	1.15 ± 0.98	
Group 6 (buccal infiltration anesthesia with lidocaine/8-min. waiting time)	2.15 ± 0.89	

Data are expressed as mean ± SD (standard deviation). Comparison between groups was made using the Kruskal-Wallis H test

A statistically significant difference was detected between the groups in terms of VAS scores measured after anesthesia (*p* = 0.019). As a result of the pairwise comparisons, there was statistically significant difference in VAS scores between the first and third groups (*p* = 0.023). The mean VAS scores in the third group were found to be statistically significantly lower than those of the first group. Additionally, there was a statistically significant difference between the fifth group and the first, second, fourth and sixth groups (*p* = 0.03, *p* = 0.015, *p* = 0.012, *p* = 0.035). The mean VAS scores in the fifth group were found to be statistically significantly lower than the first, second, fourth and sixth groups (Table 2).

**Table 2:** Distribution of VAS scores according to groups after local anesthesia

VAS Scores	Mean ± SD	P Value
Group 1 (control group with articaine)	40.00 ± 23.89	0.019*
Group 2 (buccal infiltration anesthesia with articaine/5-min. waiting time)	33.07 ± 20.56	
Group 3 (buccal infiltration anesthesia with articaine/8-min. waiting time)	22.30 ± 26.50	
Group 4 (control group with lidocaine)	37.15 ± 28.98	
Group 5 (buccal infiltration anesthesia with lidocaine/5-min. waiting time)	13.53 ± 12.68	
Group 6 (buccal infiltration anesthesia with lidocaine/8-min. waiting time)	33.23 ± 24.32	

Data are expressed as mean ± SD (standard deviation). Comparison between groups was made using the Kruskal-Wallis H test. VAS = Visual Analog Scale. *Statistically significant difference

There were no differences between the groups in terms of post-extraction Wong-Baker and VAS scores (*p* = 0.44 and *p* = 0.09) (Table 3).

**Table 3:** Distribution of Wong-Baker and VAS scores according to groups after tooth-extractions

Wong-Baker Scores	Mean ± SD	P Value
Group 1 (control group with articaine)	2.00 ± 1.08	0.44
Group 2 (buccal infiltration anesthesia with articaine/5-min. waiting time)	2.61 ± 1.19	
Group 3 (buccal infiltration anesthesia with articaine/8-min. waiting time)	1.76 ± 1.58	
Group 4 (control group with lidocaine)	1.76 ± 1.23	
Group 5 (buccal infiltration anesthesia with lidocaine/5-min. waiting time)	1.61 ± 1.38	
Group 6 (buccal infiltration anesthesia with lidocaine/8-min. waiting time)	2.30 ± 2.09	
VAS Scores		
Group 1 (control group with articaine)	36.15 ± 23.19	0.09
Group 2 (buccal infiltration anesthesia with articaine/5-min. waiting time)	46.15 ± 25.26	
Group 3 (buccal infiltration anesthesia with articaine/8-min. waiting time)	21.76 ± 29.47	
Group 4 (control group with lidocaine)	27.61 ± 24.02	
Group 5 (buccal infiltration anesthesia with lidocaine/5-min. waiting time)	24.00 ± 30.29	
Group 6 (buccal infiltration anesthesia + 8-min. waiting time with lidocaine)	40.38 ± 36.08	

Data are expressed as mean ± SD (standard deviation). Comparison between groups was made using the Kruskal-Wallis H test. VAS = Visual Analog Scale

Table 4 shows the distribution of Wong-Baker and VAS scores obtained after anesthesia and after tooth extraction in the groups that employed articaine and lidocaine as local anesthetics. There were no differences between the articaine and lidocaine groups in terms of post-anesthesia and post-extraction Wong-Baker and VAS scores.

**Table 4:** Distribution of Wong-Baker and VAS scores according to articaine and lidocaine groups after local anesthesia and tooth extractions

Wong-Baker Scores		Mean±SD	P Value
Post-anesthesia	Articaine	1.94 ± 1.25	0.984
	Lidocaine	1.94 ± 1.23	
Post-extraction	Articaine	2.12 ± 1.32	0.312
	Lidocaine	1.89 ± 1.60	
VAS Scores		Mean±SD	P Value
Post-anesthesia	Articaine	31.79 ± 24.29	0.381
	Lidocaine	27.97 ± 24.74	
Post-extraction	Articaine	34.69 ± 27.36	0.384
	Lidocaine	30.66 ± 30.56	

Data are expressed as mean ± SD (standard deviation). Comparison between groups was made using the Mann-Whitney U test. VAS = Visual Analog Scale

## Discussion

The aim of the study was to evaluate the pain experienced after anesthesia administration and in the post-extraction period in groups which had had buccal infiltration anesthesia with and without palatal anesthesia. The study’s hypothesis was accepted since there was no difference between the groups regarding post-anesthesia and post-extraction pain. This hypothesis was based on the claim that a buccally-administered local anesthetic agent may spread to the soft and hard tissues around the tooth and could eliminate the need for palatal injection [5].

Injection of local anesthesia in pediatric patients is a serious challenge and palatal injections are considered one of the most traumatic procedures [5, 18]. The palatal mucosa is tightly connected to the underlying periosteum and has an abundant nerve supply underneath. For this reason, palatal anesthesia is more painful than anesthesia applied to other areas [7]. In palatal injection, pain occurs due to the accumulation of the anesthetic in a narrow area and the displacement of the mucoperiosteum [6]. A child who experiences this kind of pain may become uncooperative and exhibit extreme dental anxiety or fear [19].

Many techniques can be used to reduce the discomfort of intraoral injections, including topical anesthetic application [20], topical cold application [21], computerized injection systems [22], pressure application, eutectic mixture of local anesthetics (EMLA) [8], and transcutaneous electronic nerve stimulation (TENS) [23]. Although different injection and auxiliary techniques have been developed, palatal injection is still a discomforting and painful procedure for patients and is not universally accepted. The literature suggests that bypassing palatal injection makes the anesthesia procedure significantly more comfortable [24]. Providing palatal anesthesia through buccal infiltration is a more feasible method than others. There are studies showing the effectiveness of this method in permanent and deciduous teeth [5]. To our knowledge, there is no study comparing the perception of anesthesia and post-extraction pain after maxillary primary molar tooth extraction in children in terms of the type of anesthesia and waiting time.

The current study found that there was no difference related to pain after anesthesia and primary molar tooth extraction between groups to which buccal anesthesia with palatal anesthesia was applied and groups to which buccal anesthesia without palatal anesthesia was applied. Uçkan et al. [6] showed in their study that painless tooth extraction can be performed in permanent maxillary cases via buccal infiltration anesthesia without palatal injection. Kolli et al. demonstrated that palatal injection could be left out in the extraction of primary maxillary molar teeth, in parallel with the results of the current study. Nevertheless they found that articaine was more effective than lidocaine during extraction with buccal infiltration anesthesia [25]. Chen et al. [26] stated in their review that a single buccal injection for primary molar extractions eliminates painful palatal injections and provides a potential advantage during treatment in pediatric patients.

The secondary aim of this study was to compare the efficacy of anesthesia with 4% articaine versus 2% lidocaine after anesthesia and tooth extraction in providing adequate palatal anesthesia with buccal injections. The present study found no difference between the efficacy of articaine and lidocaine during anesthesia and extraction. Many authors have reported that extraction of maxillary teeth is possible with buccal infiltration anesthesia without palatal injection alone, as articaine has superior anesthetic and diffusion properties, and some authors have reported similar results for lidocaine [8, 11, 27]. Bataneih et al. [5] concluded that the type of anesthetic solution did not make a difference in the perception of the pain of the injection. This may be explained by the fact that the maxilla has a porous bone structure and is thinner in children, which facilitates the diffusion of local anesthetics [27]. According to research by Bahrololoomi et al. [18], there is no difference between lidocaine and articaine when it comes to the removal of primary maxillary molar teeth, and their study attributed this outcome to the increased binding affinity of articaine to plasma proteins and the higher partition coefficient of lidocaine. Massignan et al. [28] reported no difference between articaine and lidocaine on primary maxillary molar teeth for which extraction was planned and buccal anesthesia was applied; however, they found that articaine caused more injection pain. Contrary to this study, according to research by Mittal et al. [29], articaine was insufficient for palatal anesthesia during the extraction of maxillary primary molars.

Additionally, the present study aimed to evaluate the effect of waiting time after anesthesia in terms of anesthetic agents and pain perception. It was found that a waiting period of both 5 and 8 min after buccal infiltration anesthesia was effective in anesthesia and post-extraction pain control. Also, no difference was observed in patients who were given articaine or lidocaine in terms of waiting time. This suggests that the diffusion properties of articaine and lidocaine are similar. Lima-Júnior et al. [11] suggested that with a 10-minute waiting period, a painless extraction can be performed with only buccal anesthesia. However, Uçkan et al. [6] reported that a 5-minute waiting time is sufficient for the anesthetic agent to spread. Sekhar et al. [10] reported that a waiting period of 8 min eliminated the need for palatal injection of lidocaine infiltration.

The basic factor in treating children is to manage their fear, anxiety and pain, so it is important to assess and record these conditions, using reliable and valid methods to help them communicate the level of pain they are experiencing [30, 31]. Pain can be measured using techniques such as self-report, biomarkers, and behavioral assessment, because pain is itself subjective. Self-report assessment is, however, most commonly recommended in the literature for the evaluation of pain [32]. Since perception of pain is complex and multidimensional, studies have suggested the use of more than one scale to evaluate children’s experience of pain, and have reported that both the VAS and WBS are appropriate subjective pain assessment tools [5, 31]. It is widely accepted that the WBS is suitable for assessing acute pain in children aged 3 to 18 years and the VAS in children over 8 years of age [15, 16]. Therefore, in the current study, the VAS and WBS were used together to evaluate the perception of pain after anesthesia and extraction in children between the ages of 8 and 12.

This study has some limitations. One of these is that the primary fourth and fifth teeth could have been evaluated separately. Further studies on this topic could do this. A second limitation is that objective evaluations (systolic pressure, heart rate measurement etc.) were not carried out. A third limitation is that the outcomes could be different from those of the current study in patients of different ages, particularly in young children who are uncooperative during anesthetic procedures. Consequently, including participants with a wider age range could have given us more information about the exact age limit for the application of this technique. Randomized and single or double-blinded clinical studies with larger sample populations should be conducted in future.

## Conclusion

Discomfort caused by palatal injection may preclude treating children; the authors therefore recommend that clinicians administer a single buccal infiltration of articaine or lidocaine and avoid palatal infiltration. According to the findings of the present study, extraction of primary maxillary molars without palatal anesthesia is possible with buccal infiltration of articaine or lidocaine with a waiting time of 5 or 8 min. Performing primary molar extractions with only buccal anesthesia reduces the number of injections and eliminates painful palatal injections, thus increasing the treatment compliance of pediatric patients.

## Data Availability

No datasets were generated or analysed during the current study.
